# Highly Pathogenic Avian Influenza A(H5N1) from Wild Birds, Poultry, and Mammals, Peru

**DOI:** 10.3201/eid2912.230505

**Published:** 2023-12

**Authors:** Cristopher D. Cruz, M. Eliana Icochea, Victoria Espejo, Gilda Troncos, Gina R. Castro-Sanguinetti, Megan A. Schilling, Yeny Tinoco

**Affiliations:** US Naval Medical Research Unit SOUTH, Lima, Peru (C.D. Cruz, V. Espejo, G. Troncos, M.A. Schilling, Y. Tinoco);; Universidad Nacional Mayor de San Marcos, Lima (M.E. Icochea, G.R. Castro-Sanguinetti)

**Keywords:** avian influenza, H5N1, highly pathogenic avian influenza A(H5N1), HPAI, wild birds, poultry, mammals, influenza, respiratory infections, viruses, zoonoses, Peru

## Abstract

We identified highly pathogenic avian influenza A(H5N1) virus clade 2.3.4.4b in wild birds, poultry, and a lion in Peru during November 2022–February 2023 and markers associated with transmission adaptation and antiviral drug resistance. Continuous genomic surveillance is needed to inform public health measures and avoid mass animal deaths.

Highly pathogenic avian influenza (HPAI) H5 viruses of the goose/Guangdong lineage have been categorized into multiple clades (0–9) and subclades. Viruses belonging to H5 clade 2.3.4.4 are differentiated into 8 subclades (a–h) and are of high concern because of spillover events into mammals and direct mammal-to-mammal transmission reported in Spain (*1*,*2*). HPAI H5N1 virus subclade 2.3.4.4b has been circulating in Africa, Asia, and Europe since ≈2020 (*3*,*4*). Subsequently, this subclade was identified in North America and Canada in late 2021; Colombia, Venezuela, Peru, Ecuador, and Chile during October–December 2022; and in Bolivia, Argentina, and Uruguay during January–February 2023 (*1*).

By November 2022, ≈300 dead Peruvian pelicans (*Pelecanus thagus*) and 24 dead blue-footed boobies (*Sula nebouxii*) were found on the northern coast of Peru (*5*). On November 23, 2022, the National Agrarian Health Service of Peru (Servicio Nacional de Sanidad Agraria del Peru) and the US Naval Medical Research Unit SOUTH reported HPAI H5N1 virus was present in Peru (*6*). Subsequently, we sequenced 18 additional virus samples positive for hemagglutinin (HA) subtype 5 (H5) that were collected from 3 Peruvian pelicans, 12 chickens (*Gallus gallus domesticus*), 2 Neotropic cormorans (*Nannopterum brasilianum*), and 1 lion (*Panthera leo*, from a zoo). We extracted viral RNA from respiratory tissue or environmental fecal samples. We collected samples from birds during November–December 2022 from northern and central coasts of Peru and the sample from the lion in February 2023 from Junin (Andean region) (Appendix Table 1).

We amplified influenza A virus genomes by using a modified protocol (*7*). We prepared libraries by using the Nextera XT DNA Library Preparation Kit (Illumina, https://www.illumina.com) and sequenced them by using the MiSeq Reagent Kit v3 (600-cycle paired-end) on the MiSeq platform (Illumina). We trimmed raw reads, removed host sequences, and then de novo assembled the filtered reads. We identified the resulting contigs as H5N1 by using a BLASTn search (https://blast.ncbi.nlm.nih.gov). We deposited all obtained sequences in GenBank (accession nos. OQ547312–451).

We performed phylogenetic analysis to classify subclades by using the maximum-likelihood method. We retrieved H5 sequences from HPAI clade 2.3.4.4 and low pathogenicity avian influenza viruses published in GISAID (https://www.gisaid.org) and GenBank during 2014–2023 (until July 20, 2023). The phylogenetic tree of HA sequences placed H5N1 strains from North, Central, and South America into different groups within subclade 2.3.4.4b. We identified 6 subclades comprising sequences from 1–5 countries (Venezuela, Colombia, Ecuador, Mexico/Honduras/Costa Rica/Panama/Colombia, Costa Rica/Panama/Colombia, and Ecuador/Peru/Chile) and 1 sequence from Colombia that did not cluster with other strains from South America. Our results suggest that the strains from South America were not monophyletic and represented 7 independent virus introduction events (Figure), complementing a previous report (*8*).

**Figure:**
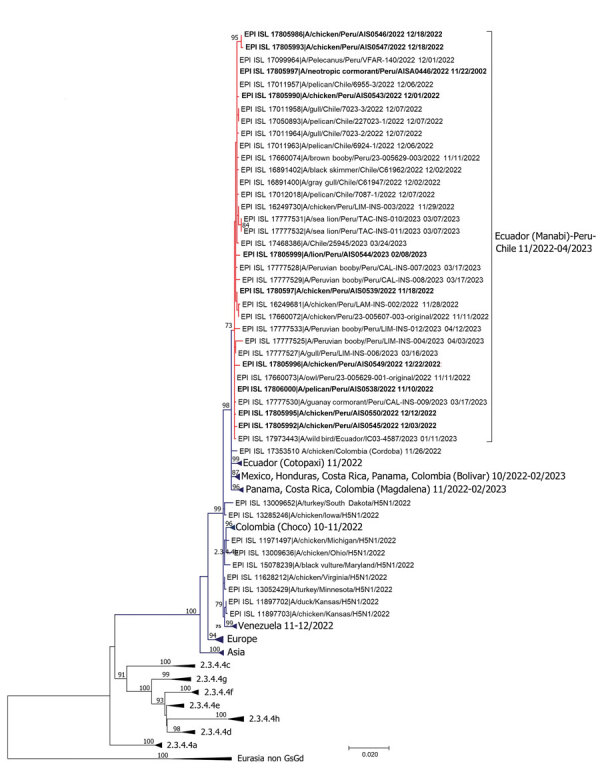
Phylogenetic analysis of highly pathogenic avian influenza A(H5N1) from wild birds, poultry, and mammals, Peru. Maximum-likelihood method was used for phylogeny of 101 hemagglutinin H5 sequences from avian influenza viruses. Red lines indicate clustering of strains from Peru and sequences from this study; bold font indicates the sequences from this study. Dark blue lines indicate other strains from South and North America. Non–goose/Guangdong lineage virus strains from Eurasia were outgroups. Phylogenetic tree was generated and edited with MEGA X software (https://www.megasoftware.net). Sequences were aligned by using the MUSCLE program in the AliView alignment viewer and editor (https://www.ormbunkar.se/aliview). We used general time reversible and gamma distribution models; robustness of tree topology was assessed with 1,000 bootstrap replicates. Only bootstrap values >70% are shown. Scale bar indicates nucleotide substitutions per site.

We also compared available amino acid sequences of virus proteins among strains from South America to identify differences among subclades (Appendix Table 2). We identified several amino acid changes that were shared among members of the same subclade (Appendix Table 3). Those changes were consistent with our HA phylogenetic analysis, supporting the hypothesis that independent virus introduction events occurred in South America. 

We performed molecular marker analysis to identify specific amino acid mutations associated with HPAI adaptation, transmission, and antiviral drug resistance, such as those in neuraminidase (NA), matrix protein 2, and polymerase acidic protein (*9*). We identified 21 molecular markers involved in HPAI H5N1 pathogenicity that were present in all analyzed sequences from South America and 7 markers that were found in some sequences (Table). However, 2 mutations in the polymerase basic 2 protein (Q591K and D701N) associated with mammal adaptation were identified only in sequences from sea lions in Peru and from 1 human case in Chile. The T271A mutation in polymerase basic 2 protein linked to mammal adaptation and S369I and I396M mutations in NA that were observed in the mink outbreak in Spain (*2*) were not found in sequences from South America. We did not find amino acid mutations related to resistance to the antiviral drugs oseltamivir, zanamivir and peramivir (in NA), amantadine and rimantadine (in matrix protein 2), or baloxavir (in PA). We only found the H252Y mutation in NA associated with moderately reduced susceptibility to oseltamivir (*10*).

**Table:** Summary of molecular markers identified in influenza virus strains from South America in study of highly pathogenic avian influenza A(H5N1) from wild birds, poultry, and mammals, Peru*

Protein	Mutation/motif	Phenotype
PB2	D9N†	Increases virulence in mice
	L89V, G309D, T339K, R477G, I495V, K627E, A676T	Increases polymerase activity in mammalian cell lines and increases virulence in mice
	Q591K‡	Increases polymerase activity in mammalian and avian cell lines, increases replication in mammalian cell lines, increases virulence in mice
	D701N‡	Increases polymerase activity, enhances replication efficiency, increases virulence and contact transmission in guinea pigs, increases virulence in mice
PB1	D3V	Increases polymerase activity and viral replication in avian and mammalian cell lines
	D622G	Increases polymerase activity and virulence in mice
PB1-F2	N66S	Enhances replication, virulence, and antiviral response in mice
PA	N383D	Increases polymerase activity in mammalian and avian cell lines
HA	D94N,§ S133A, S154N	Increases virus binding to α2–6 receptor
	T156A	Increases virus binding to α2–6, increases transmission in guinea pigs
	S107R, T108I	Increases virulence in chickens and mice and the pH of fusion
	K218Q, S223R	Increases virus binding to α2–3 and α2–6 receptors
	321-329 (PLR(EorG)KRRKR)	Polybasic cleavage motif sequence required for HPAIV
NP	M105V¶	Increases virulence in chickens
	I109T#	Increases polymerase activity and viral replication in chickens (but not ducks), increases virulence in chickens
	A184K	Increases replication in avian cells and virulence in chickens
M1	N30D	Increases virulence in mice
	I43M	Increases virulence in mice, chickens and ducks
	T215A	Increases virulence in mice
M2	I27A**	Increases resistance to amantadine and rimantadine
NS1	P42S	Increases virulence and decreases the antiviral response in mice
	C138F	Increases replication in mammalian cell and decreases the interferon response
	V149A	Increases virulence and decreases the interferon response in chickens
	L103F, I106M	Increases virulence in mice
	K55E, K66E, C138F	Enhances replication in mammalian cells and decreases the interferon response

*Molecular markers of influenza virus strains were identified as previously described (*9*). HA, hemagglutinin; HPAIV, highly pathogenic avian influenza virus; M1, matrix protein 1; M2, matrix protein 2; NP, nucleoprotein; NS1, nonstructural protein 1. †Only in 2 sequences from pelicans (GISAID [https://www.gisaid.org] accession nos. EPI_ISL_17099964, EPI_ISL_17165223). ‡Only in sequences from 2 sea lions in Peru and 1 human case in Chile. §Only in 1 sequence from a wild bird in Peru (GISAID accession no. EPI_ISL_17660074). ¶Mutation sequences from Venezuela and Colombia (Choco) have M rather than V. #Only in sequences from Colombia (Choco). **Only in 1 sequence from a wild bird in Peru (GISAID accession no. EPI_ISL_17777528).

In conclusion, HPAI H5N1 virus clade 2.3.4.4b was identified in samples collected in Peru from wild birds, poultry, and a lion during November 2022–February 2023. According to phylogenetic analysis, the multiple cluster distribution revealed independent introductions of HPAI H5N1 clade 2.3.4.4b viruses into South America from North and Central America. Four introductions occurred in Colombia, 2 in Ecuador, and 1 in Venezuela/Peru. In addition, strains from Peru were closely related to those from Ecuador and Chile. Finally, we describe the presence of previously reported mutations that might have public health implications because of their associations with increased virulence and virus replication and mammal host adaptation along with reduced susceptibility to oseltamivir. Continuous genomic surveillance is needed to identify markers associated with mammal adaptation and potential human-to-human transmission, to inform public health measures, avoid mass animal deaths, and to protect human populations.

Appendix Additional information for highly pathogenic avian influenza A(H5N1) from wild birds, poultry, and mammals, Peru
